# Clinically detected hepatitis B virus reactivation in HBsAg-negative, anti-HBc-positive breast cancer patients receiving systemic therapy without antiviral prophylaxis: a two-centre descriptive cohort and nested case study

**DOI:** 10.3389/fonc.2026.1908493

**Published:** 2026-07-27

**Authors:** Göknur Yapar Toros, Sabahat Çeken, Ayşe Ocak Duran, Öztürk Ateş

**Affiliations:** 1 Department of Infectious Diseases and Clinical Microbiology, Ankara Etlik City Hospital, Ankara, Türkiye; 2 Department of Medical Oncology, Ankara Dr. Abdurrahman Yurtaslan Oncology Training and Research Hospital, Ankara, Türkiye

**Keywords:** anthracyclines, anti-HBc–positive, anti-HBs, antiviral prophylaxis, breast cancer chemotherapy, hepatitis B reactivation, resolved HBV infection

## Abstract

**Background:**

Hepatitis B virus (HBV) reactivation is a recognised complication in HBsAg-negative, anti-HBc-positive patients receiving cytotoxic cancer therapy. Guidelines place anti-HBc-positive patients exposed to anthracyclines in a moderate-risk band warranting on-treatment monitoring or selective prophylaxis, yet real-world data from non-prophylaxis breast cancer practice, where serial HBV DNA surveillance is often incomplete, remain scarce.

**Methods:**

We retrospectively studied HBsAg-negative, anti-HBc-positive breast cancer patients treated with systemic therapy between 2018 and 2024 at two centres in Turkey, none of whom received antiviral prophylaxis. We extracted demographic, oncological and virological data, including baseline anti-HBs titres and all available HBV DNA and alanine aminotransferase (ALT) results, and we quantified the completeness of virological surveillance. Because on-treatment HBV DNA was tested only when hepatitis was clinically suspected, the outcome captured was clinically detected reactivation; the study therefore describes surveillance quality rather than estimating true incidence. With a single event, analyses were kept descriptive.

**Results:**

Among 139 patients (mean age 60.1 years), 89.2% were anti-HBs-positive and 65.5% received anthracyclines. Baseline HBV DNA was documented in all patients, but serial on-treatment monitoring was not. One clinically overt reactivation occurred (0.7%; 95% CI 0.02%–3.94%) in an anti-HBs-negative woman receiving doxorubicin–cyclophosphamide, presenting as icteric hepatitis at month 4 and responding to entecavir. Since subclinical events would have escaped detection, this figure is best read as a lower bound rather than a true incidence.

**Conclusions:**

The single reactivation we detected arose in the patient profile that the guidelines flag as moderate-risk—absent anti-HBs together with anthracycline exposure. These observations are consistent with, but do not establish, a risk-adapted approach, given one event and incomplete surveillance. Prospective studies using standardised serial HBV DNA monitoring are needed to estimate true reactivation incidence in this population.

## 1 Introduction

Hepatitis B virus (HBV) reactivation is a clinically important complication of cytotoxic and immunosuppressive therapy that can cause acute hepatitis, hepatic decompensation and interruption of anticancer treatment. During immunosuppression, viral replication may rise before biochemical hepatitis becomes apparent, whilst immune reconstitution after treatment can precipitate hepatocellular injury. Although reactivation has been most extensively characterised in haematological malignancies and with B cell-depleting therapy, it is also recognised across solid tumours as systemic therapy has expanded. Clinically meaningful reactivation can occur not only in chronic carriers but also in patients with resolved infection (HBsAg-negative, anti-HBc-positive), reflecting the persistence of intrahepatic covalently closed circular DNA and the potential for renewed replication under immune pressure (1, 2).

A recent systematic review and meta-analysis (86 studies, 21,297 patients) reported a pooled HBV reactivation rate of approximately 9% across malignancies—approximately 10% in haematological and 5% in solid tumours, with lower pooled rates in the anti-HBc-positive and anti-HBs-positive subgroups (3). Contemporary guidance stratifies management by HBV serostatus and the type and intensity of immunosuppression: antiviral prophylaxis is recommended for high-risk exposures, whereas close virological monitoring or selective prophylaxis is endorsed for intermediate (moderate)-risk regimens, within which anti-HBc-positive patients exposed to anthracyclines are placed (1, 4).

Breast cancer is a relevant setting because anthracycline-containing chemotherapy is widely used. Two further factors modulate this risk: absent or low anti-HBs antibody, which has been associated with a higher likelihood of reactivation amongst anti-HBc-positive patients (4), and the completeness of virological surveillance, as reliance on alanine aminotransferase (ALT) alone may miss subclinical reactivation detectable only by serial HBV DNA (5–7).

In this routine-care cohort, we obtained on-treatment HBV DNA testing only when hepatitis was clinically suspected; the study therefore captures clinically detected reactivation and characterises the completeness of virological surveillance (a healthcare-delivery question) rather than estimating true incidence. We also deliberately excluded patients who received antiviral prophylaxis so that the natural non-prophylaxis course could be described; because prophylaxis is preferentially offered to higher-risk patients, this concentrates the cohort towards lower-risk individuals, a selection bias we acknowledge at the outset.

Real-world descriptions of how reactivation manifests in breast cancer patients with resolved infection treated without prophylaxis (and of how often virological surveillance is actually performed in routine care) remain limited. We therefore conducted a two-centre descriptive cohort study, complemented by a detailed nested case study, with three explicitly descriptive objectives: 1) to characterise the completeness and triggers of HBV DNA and ALT surveillance in this setting, 2) to document the frequency of clinically detected HBV reactivation under routine non-prophylaxis practice as a consequence of that surveillance and 3) to describe, in depth, the clinical, biochemical and virological course of any reactivation event. Because we anticipated a few events, the study was designed to be descriptive and hypothesis-generating rather than to test risk factors.

## 2 Methods

This was a retrospective, two-centre descriptive cohort study with a nested case study, evaluating HBV reactivation amongst HBsAg-negative, anti-HBc-positive patients with breast cancer undergoing systemic anticancer therapy. Patients were identified from two tertiary oncology centres in Ankara, Turkey—Ankara Dr. Abdurrahman Yurtaslan Oncology Training and Research Hospital and Ankara Etlik City Hospital—between 2018 and 2024. All data were obtained from existing electronic medical records without additional diagnostic or interventional procedures. Reporting follows the Strengthening the Reporting of Observational Studies in Epidemiology (STROBE) recommendations for observational studies.

Adults (≥18 years) with histologically confirmed breast cancer who were 1) HBsAg-negative and anti-HBc-positive at baseline, 2) had a documented anti-HBs result and 3) received at least one cycle of systemic chemotherapy or targeted therapy were included. Patients who received antiviral prophylaxis were excluded so that the natural, non-prophylaxis course could be described. Because prophylaxis is preferentially offered to patients judged highest-risk, this exclusion concentrates the cohort towards lower-risk individuals; the resulting frequency therefore does not represent the risk of the broader anti-HBc-positive breast cancer population. During the study period, 160 anti-HBc-positive breast cancer patients were screened; 21 received antiviral prophylaxis and were excluded, leaving 139 non-prophylaxis patients for analysis. Thus, the analytic cohort represented 86.9% of the screened anti-HBc-positive population.

Extracted variables comprised age; tumour stage, grade and histology; and exposure to anthracyclines, taxanes, HER2-directed agents, CDK4/6 inhibitors, platinum compounds and other cytotoxics. HBV-related variables included baseline anti-HBs status and quantitative titre, baseline HBV DNA, any on-treatment or post-treatment HBV DNA results, HBV serology at suspected reactivation, ALT/aspartate aminotransferase (AST) and follow-up duration.

At both centres, baseline HBV screening (HBsAg, anti-HBc total/IgG and anti-HBs) was performed routinely before systemic therapy. During the study period, there was no standardised institutional protocol mandating fixed-interval serial HBV DNA monitoring for HBsAg-negative, anti-HBc-positive patients who did not receive prophylaxis. On-treatment virological surveillance was therefore *reactive* (clinically triggered) rather than *prospective* (serial, fixed-interval): ALT was obtained as part of routine pre-cycle laboratory assessment during chemotherapy, whereas HBV DNA was not scheduled and was ordered only when hepatitis was clinically suspected (ALT elevation or symptoms). To make this design measurable, for every patient, three pre-specified ascertainment metrics were recorded: whether baseline HBV DNA was available, whether any on-treatment HBV DNA was obtained and whether ALT was monitored during therapy (**Table 1**). This pragmatic context—universal baseline serology but clinically triggered rather than protocolised virological follow-up—is central to the interpretation of our findings.

**Table 1:** Completeness and triggers of HBV virological surveillance (N = 139).

Surveillance element	n (%)	Comment
Baseline HBsAg/anti-HBc/anti-HBs documented	139 (100)	Universal pre-treatment screening
Baseline HBV DNA documented	139 (100)	Undetectable in 138; low-level in 1
Protocolised fixed-interval serial HBV DNA on therapy	0 (0)	No standardised monitoring protocol
On-treatment HBV DNA obtained (ALT/symptom-triggered)	1 (0.7)	Only in the reactivation case
ALT monitored during therapy (intermittent)	139 (100)	Part of routine oncological follow-up
Clinically detected reactivation	1 (0.7)	Overt icteric hepatitis, month 4

Reactivation without ALT elevation could not be ascertained, so the detected rate is a floor on the true frequency.

HBV, hepatitis B virus; ALT, alanine aminotransferase.

HBV reactivation was defined, in accordance with American Society of Clinical Oncology (ASCO) 2020, American Gastroenterological Association (AGA) 2025 and European Association for the Study of the Liver (EASL) 2025, as any of 1) reappearance of detectable HBV DNA in a patient with previously undetectable HBV DNA, 2) a ≥2 log_10_ (≥100-fold) rise in HBV DNA from baseline, or 3) HBsAg seroreversion (negative to positive), with or without biochemical hepatitis (ALT elevation) (1, 4, 8).

### 2.1 Statistical analysis

Analyses were pre-specified as descriptive. Continuous variables are summarised as mean ± standard deviation and median [interquartile range (IQR)] where distributions were skewed; categorical variables are summarised as counts and percentages. The completeness of virological surveillance was pre-specified as a secondary outcome, operationalised through the three binary ascertainment metrics defined in the Methods section and reported in **Table 1**. The detected event rate is presented with an exact (Clopper–Pearson) 95% confidence interval, which reflects sampling uncertainty around the observed rate rather than the true biological incidence. Because only one reactivation event was observed, between-group hypothesis testing, multivariable modelling or risk-factor estimation was not performed; any apparent differences between subgroups were reported descriptively and should not be read as measures of reactivation risk.

## 3 Results

We present the surveillance-ascertainment findings first because they frame how the reactivation frequency should be read. Baseline HBV DNA was available for all 139 patients (100%) and baseline anti-HBs for all 139 (100%). In contrast, no patient underwent protocol-driven, fixed-interval serial HBV DNA monitoring during therapy; on-treatment HBV DNA was obtained only when hepatitis was clinically suspected (ALT- or symptom-triggered). ALT was monitored at least intermittently in routine oncological follow-up (**Table 1**). This pattern means that a reactivation without ALT elevation would have gone unnoticed; the case we describe was caught precisely because it presented as overt icteric hepatitis. The 0.7% figure should therefore be read as a floor, not an estimate of the true rate in this cohort.

The cohort comprised 139 anti-HBc-positive breast cancer patients (mean age 60.1 ± 10.7 years; range 34–88). At baseline, 124 patients (89.2%) were anti-HBs-positive (mean titre 278.0 ± 378.4 IU/L; range 10–1,000), and 15 (10.8%) were anti-HBs-negative. Baseline HBV DNA was undetectable in 138 patients (99.3%) and low-level detectable in 1 patient (0.7%). Follow-up averaged 11.1 ± 6.7 months (range 1–32); given the skew, the median follow-up was 10.0 months [IQR 6.0–15.0]. Most patients had early-stage (43.2%) or locally advanced (30.9%) disease; tumour grade and histology were unrecorded in the majority of source records (74% and 78%, respectively) and were not analysed further. Anthracycline-based regimens were administered to 91 patients (65.5%), taxanes to 103 (74.1%), HER2-directed therapy to 19 (13.7%), CDK4/6 inhibitors to 7 (5.0%) and platinum to 5 (3.6%) (**Table 2**).

**Table 2:** Baseline demographic, virological, tumour and treatment characteristics (N = 139).

Characteristic	Category	n	%/Mean ± SD
Age (years)	Mean ± SD (range)	139	60.1 ± 10.7 (34–88)
Anti-HBs status	Negative	15	10.8
	Positive	124	89.2
Anti-HBs titre (IU/L)	Mean ± SD (range)	124	278.0 ± 378.4 (10–1000)
Follow-up (months)	Mean ± SD (range)	139	11.1 ± 6.7 (1–32)
Follow-up (months)	Median [IQR]	139	10.0 [6.0–15.0]
Baseline HBV DNA	Undetectable	138	99.3
	Detectable (low-level)	1	0.7
Cancer stage	Early (I–II)	60	43.2
	Locally advanced (III)	43	30.9
	Metastatic (IV)	8	5.8
	Unknown	28	20.1
Anthracycline regimen	Yes	91	65.5
Taxane regimen	Yes	103	74.1
HER2-directed therapy	Yes	19	13.7
CDK4/6 inhibitor	Yes	7	5.0
Platinum-based regimen	Yes	5	3.6
Other cytotoxic agents	Yes	27	19.4
HBV reactivation	Yes	1	0.7

Percentages for treatment exposures are not mutually exclusive. Anti-HBs titre summarised amongst the 124 anti-HBs-positive patients. Tumour grade and histology were unrecorded in 74% and 78% of cases, respectively, and are omitted as non-informative.

IQR, interquartile range; HBV, hepatitis B virus.

The single patient with low-level detectable baseline HBV DNA was distinct from the reactivation case (whose baseline HBV DNA was undetectable); both fell in the anthracycline group. This patient was managed without prophylactic antiviral therapy, with routine ALT-based oncological follow-up and HBV DNA testing only if hepatitis was clinically suspected, and did not develop clinically detected reactivation during the available follow-up.

These descriptive differences are presented for transparency only and are not risk estimates. Descriptively, anthracycline-exposed patients were younger (mean 58.1 vs. 63.8 years) and had longer recorded follow-up (12.5 vs. 9.1 months) and higher anti-HBs titres (median 127 vs. 10 IU/L) than non-exposed patients; anti-HBs status, baseline HBV DNA and stage distributions were broadly similar. The single reactivation occurred in the anthracycline group and in the anti-HBs-negative subgroup (**Table 3**).

**Table 3:** Descriptive characteristics by anthracycline exposure, with event distribution by baseline anti-HBs status (consolidated; no inferential testing performed).

Characteristic	Anthracycline (−) n = 48	Anthracycline (+) n = 91
Age, years—mean ± SD	63.8 ± 10.9	58.1 ± 10.0
Follow-up, months—mean ± SD	9.1 ± 5.4	12.5 ± 7.2
Anti-HBs titre, IU/L—median [IQR]	10 [172]	127 [568]
Anti-HBs negative—n (%)	3 (6.3)	12 (13.2)
Baseline HBV DNA detectable—n (%)	0 (0)	1 (1.1)
Stage I–II—n (%)	21 (43.8)	39 (42.9)
Stage III—n (%)	10 (20.8)	33 (36.3)
Stage IV—n (%)	5 (10.4)	3 (3.3)
Stage unknown—n (%)	12 (25.0)	16 (17.6)
HBV reactivation—n (%)	0 (0)	1 (1.1)

Event distribution by baseline anti-HBs status (previously tabulated separately): anti-HBs-negative 1/15, anti-HBs-positive 0/124 and total 1/139. Anti-HBs titres are summarised amongst patients with available quantitative titres; values <10 IU/L were classified as negative. Descriptive proportions only—with a single event, no comparative inference can be drawn.

IQR, interquartile range.

The former **Tables 3**, **4** have been consolidated into a single descriptive table (**Table 3**) to avoid over-interpreting a single event and to improve readability.

**Table 4:** Detailed clinical and virological profile of the single reactivation case.

Parameter	Value
Age/sex	61 years/female
Diagnosis	Invasive ductal breast carcinoma, grade 2
Regimen	4 cycles doxorubicin + cyclophosphamide (anthracycline-based)
Baseline HBsAg/anti-HBc/anti-HBs	Negative/positive/negative (<10 IU/L)
Baseline HBV DNA	Undetectable
Time to reactivation	Month 4 (from chemotherapy initiation)
Reactivation findings	ALT 1533, AST 1247 U/L; GGT 267, ALP 219 U/L; total bilirubin 7.2 mg/dL; clinical jaundice; HBV DNA 6.9 × 10^7^ copies/mL
Serology at reactivation	HBsAg seroreversion (positive); anti-HBc IgM positive; HBeAg negative/anti-HBe positive
Antiviral therapy	Entecavir
HBV DNA kinetics (after entecavir initiation)	Month 1: 4.3 × 10^5^ → month 3: 8.5 × 10^3^ → month 6: undetectable (copies/mL); month 6 corresponds to chemotherapy month 10
Outcome	Biochemical normalisation; HBsAg clearance by month 10; anti-HBs did not develop

ALT, alanine aminotransferase.

Compared with external pooled estimates of approximately 5% in solid tumours and 4% in anti-HBc-positive patients (3), our clinically detected rate of 0.7% is several-fold lower, which is consistent with under-ascertainment under ALT-triggered surveillance. This comparison should be interpreted only as contextual evidence, not as a formal estimate of detection sensitivity. In the sensitivity analysis restricted to patients with ≥3 months of follow-up (n = 132), clinically detected HBV reactivation occurred in one patient (0.8%; exact 95% CI 0.02%–4.15%). The single reactivation, detected at month 4, remained within this restricted cohort, and the interpretation was unchanged: the detected rate remained a lower bound because HBV DNA testing was clinically triggered rather than protocolised.

### 3.1 Nested case study of the single reactivation event

A 61-year-old woman with invasive ductal breast carcinoma (grade 2) was evaluated before adjuvant chemotherapy (**Table 4**). Baseline virology showed HBsAg-negative, anti-HBc IgG-positive and anti-HBs <10 IU/L (resolved HBV infection without protective antibody) with normal liver enzymes and undetectable HBV DNA. In keeping with local practice at the time, no antiviral prophylaxis was given.

She received four cycles of doxorubicin–cyclophosphamide (an anthracycline-based, moderate-risk regimen). Approximately 4 months after starting chemotherapy, she developed acute jaundice and constitutional symptoms. Investigations showed marked hepatocellular injury (ALT 1,533 U/L, AST 1,247 U/L), cholestasis (gamma-glutamyl transferase (GGT) 267 U/L, alkaline phosphatase (ALP) 219 U/L) and hyperbilirubinaemia (total bilirubin 7.2 mg/dL). HBV serology demonstrated HBsAg seroreversion with anti-HBc IgM positivity and HBeAg negativity, and quantitative HBV DNA was 6.9 × 10^7^ copies/mL, confirming overt reactivation.

**Figure 1:**
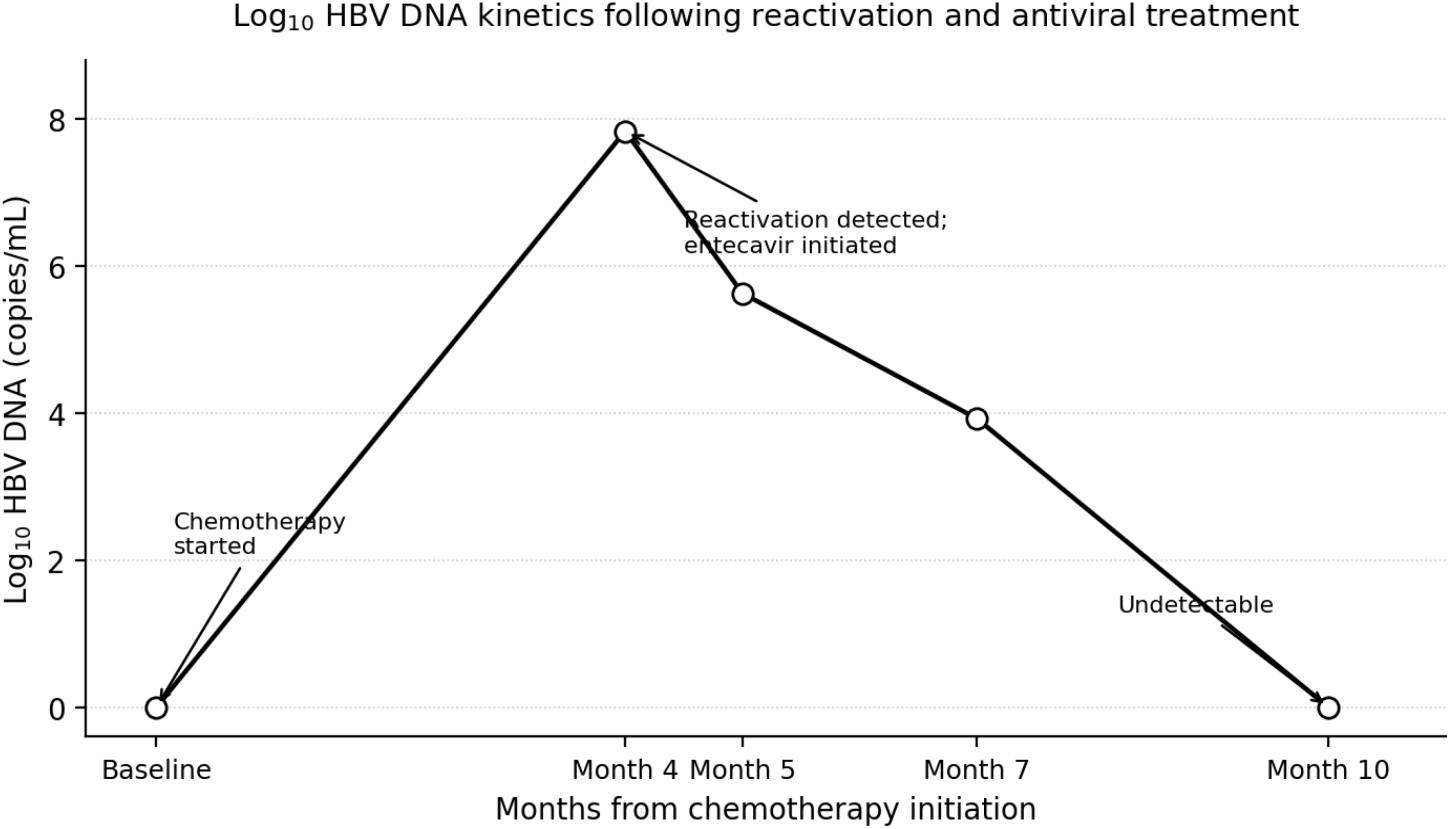
Log_10_ HBV DNA kinetics (copies/mL) in the single reactivation case. Time is shown on a single axis as months from chemotherapy initiation. Reactivation was detected at month 4 (6.9 × 10^7^ copies/mL); entecavir was started immediately, and HBV DNA became undetectable by month 10 (i.e. 6 months of entecavir).

Entecavir was started promptly. HBV DNA fell to 4.3 × 10^5^ copies/mL after 1 month of entecavir and 8.5 × 10^3^ copies/mL after 3 months and became undetectable after 6 months of therapy—corresponding to chemotherapy month 10 (**Figure 1**). Follow-up at month 10 confirmed HBsAg clearance, although protective anti-HBs did not develop; the patient remained clinically stable. This single event occurred in a patient combining the two features that current guidelines flag as moderate-risk—absent anti-HBs and anthracycline exposure—together with the absence of prophylaxis. It is presented as an illustrative case and, by itself, cannot establish causal risk attribution.

## 4 Discussion

In this two-centre cohort of 139 HBsAg-negative, anti-HBc-positive breast cancer patients treated without prophylaxis, one overt HBV reactivation was detected (0.7%). This single event should not be interpreted as a precise incidence estimate; rather, the study highlights that the event occurred in a patient with features consistent with guideline-defined moderate risk (no protective anti-HBs, anthracycline exposure) and that it was detected only because the hepatitis was severe enough to prompt testing.

Our surveillance metrics (**Table 1**) show universal baseline virology but no protocolised serial HBV DNA monitoring during therapy. Prospective breast cancer data have shown that serial HBV DNA testing markedly increases diagnostic sensitivity and that viral replication can rise before ALT does (5–7). Where HBV DNA is checked only after ALT rises, reactivation that stays subclinical will be missed. Consistent with this, our clinically detected 0.7% sits well below pooled reactivation estimates of approximately 5% for solid tumours and 4% in anti-HBc-positive patients (3), reinforcing that the true frequency is likely higher.

Current frameworks classify HBsAg-negative, anti-HBc-positive patients exposed to anthracyclines as moderate-risk, for whom guidelines endorse either on-treatment HBV DNA/ALT monitoring at 1–3-month intervals or selective antiviral prophylaxis rather than universal prophylaxis (1). ASCO 2020 similarly recommends baseline HBsAg, anti-HBc and anti-HBs testing and notes that absent anti-HBs identifies patients at higher reactivation risk (4). Our single case is fully consistent with this stratification; we are careful, however, not to over-read one event—consistency with a framework is not independent confirmation of it.

Among anti-HBc-positive patients, low or absent anti-HBs is consistently associated with higher reactivation risk, and a meta-analysis of breast cancer chemotherapy cohorts found reactivation clustering in patients with low or absent anti-HBs on more intensive regimens (9). Low or absent anti-HBs may indicate reduced humoral immune control over residual intrahepatic cccDNA and has been associated with higher reactivation risk in prior cohorts. Our one event occurred in the anti-HBs-negative subgroup—an observation in keeping with this literature, although a single case cannot demonstrate a prognostic role on its own.

The universal baseline screening achieved at both centres is a strength, but it also limits generalisability. A recent meta-analysis of pre-chemotherapy HBV screening found a pooled screening rate of only approximately 57%, falling to roughly 37% in solid-tumour patients versus 65% in haematological malignancies, and ranging from approximately 89% in high-endemic countries to 49% in low-endemic countries (10). Against this backdrop, our 100% baseline screening represents a best-case scenario that is not representative of routine oncology practice. It also implies that in real-world settings, where a substantial share of patients are never screened, the true burden of undetected reactivation may be greater than that captured in screened, non-prophylaxis cohorts.

Hepatitis during chemotherapy is not always viral; cytotoxic agents can produce hepatic patterns independent of HBV replication (11). In our case, concurrent HBsAg seroreversion, anti-HBc IgM positivity and a high HBV DNA load unambiguously identified reactivation rather than drug injury, underscoring why virological confirmation—not ALT alone—is needed to classify on-treatment hepatitis correctly and to time antiviral therapy.

### 4.1 Strengths and limitations

The study describes a real-world, non-prophylaxis cohort with universal baseline virology, an explicit accounting of surveillance completeness and a virologically confirmed, longitudinally characterised reactivation case. Several limitations constrain interpretation. First, virological surveillance was not standardised; the absence of protocolised serial HBV DNA means that subclinical reactivation was likely missed, so 0.7% is a lower bound, not a true incidence. Second, only one event occurred, precluding any risk-factor inference. Third, follow-up was short and heterogeneous (range 1–32 months); because reactivation can occur during therapy and for 6–12 months afterwards, patients with only a few months of follow-up cannot be considered reliably reactivation-free, which further inflates apparent safety and compounds under-ascertainment. Fourth, the exclusion of prophylaxis patients is a major source of selection bias, concentrating the cohort towards lower-risk individuals; the 139-patient analytic cohort represented 86.9% of the 160 screened anti-HBc-positive patients (21 excluded for prophylaxis) and should be interpreted specifically as a selected non-prophylaxis sample rather than as the full screened anti-HBc-positive breast cancer population. Fifth, tumour grade and histology were missing in roughly three-quarters of records. Finally, newer agents (e.g. immune checkpoint inhibitors and antibody–drug conjugates) were rare or absent, so nothing can be inferred about their reactivation risk.

## 5 Conclusion

In a non-prophylaxis, two-centre cohort of HBsAg-negative, anti-HBc-positive breast cancer patients, the one HBV reactivation we detected occurred in a woman who lacked protective anti-HBs and received an anthracycline—the combination guidelines treat as moderate-risk. Because virological testing was triggered by clinical hepatitis rather than performed routinely, and follow-up was short for some patients, the 0.7% figure understates the true frequency. We therefore read these data not as an incidence estimate but as a real-world illustration of a surveillance gap that supports anti-HBs-informed screening and structured HBV DNA monitoring. Confirming the true incidence will require prospective cohorts with standardised serial surveillance.

## Data Availability

The raw data supporting the conclusions of this article will be made available by the authors, without undue reservation.
